# Supplementary figures and images for: DHA but not EPA induces the trans-differentiation of C2C12 cells into white-like adipocytes phenotype
Source: PLoS One. 2021 Sep 2;16(9):e0249438. doi: 10.1371/journal.pone.0249438 (PMC8412409; doi:10.1371/journal.pone.0249438)

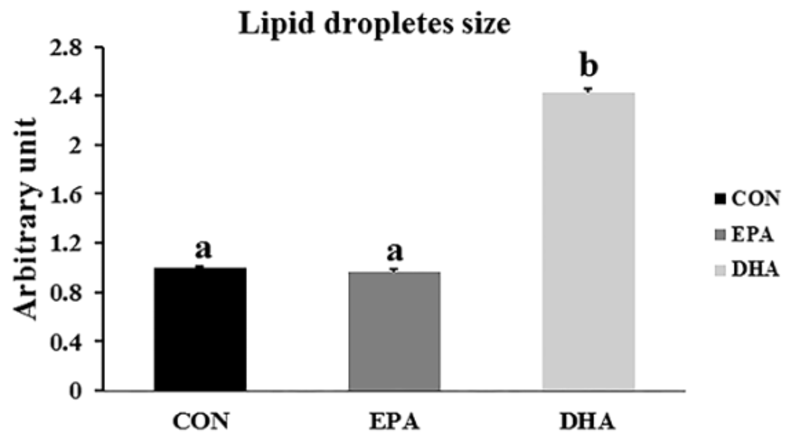

Supplement: S1 Fig — The cells were treated with differentiation induction medium (DIM) in the absence (CON) or presence of isolated doses of 50 μM EPA and 50 μM DHA for 7 days. Significant differences among groups were measured in arbitrary unit and presented as mean ± SEM. Different letters indicate significant differences between treatment groups where b = P < 0.05; n = 10. (TIF) [file pone.0249438.s001.tif]

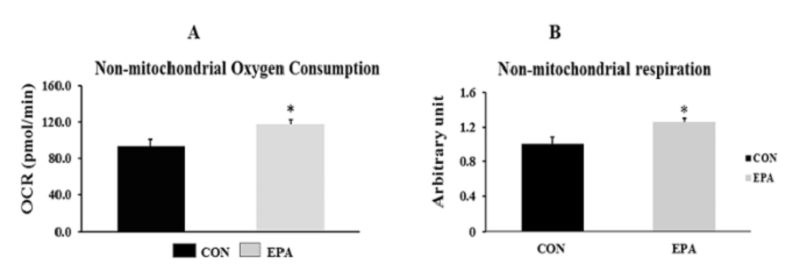

Supplement: S2 Fig — (A) the effect of EPA treatment on non- mitochondrial respiration. OCR traces are expressed as pmol O2 per min in C2C12 cells and normalized to protein concentration (B). The Relative differences in non- mitochondrial respiration of control and EPA treated group. Significant differences among groups were measured in arbitrary unit and presented as mean ± SEM. *P < 0.05; n = 3; 12 measurements. (TIF) [file pone.0249438.s002.tif]
