# Supplementary material for: DHA but not EPA induces the trans-differentiation of C2C12 cells into white-like adipocytes phenotype
Source: PLoS One. 2021 Sep 2;16(9):e0249438. doi: 10.1371/journal.pone.0249438 (PMC8412409; doi:10.1371/journal.pone.0249438)
Supplement: S1 Table — (DOCX) [file pone.0249438.s003.docx]

**Supplemental table 1: size of lipid droplets in C2C12 cells induced to brown adipogenesis***

| Group | Sample 1 | Sample 2 | Sample 3 | Sample 4 | Sample 5 | Sample 6 | Sample 7 | Sample 8 | Sample 9 | Sample 10 | STD |
| --- | --- | --- | --- | --- | --- | --- | --- | --- | --- | --- | --- |
| CON | 3186.268 | 3354.850 | 3236.355 | 3204.561 | 3206.965 | 3169.153 | 3345.820 | 3169.775 | 2869.833 | 3264.282 | 45.13251 |
| EPA | 2698.239 | 3511.440 | 3200.754 | 2928.734 | 3144.735 | 3120.469 | 3355.427 | 2895.679 | 3167.165 | 2932.229 | 75.42789 |
| DHA | 7743.504 | 7684.104 | 7278.146 | 7769.445 | 7998.223 | 8538.897 | 7659.444 | 8156.365 | 7336.790 | 7829.387 | 117.2521 |

***** The size was measured in nanometer. Scale bar is 100µm. The value of each sample represents the mean of 10 measurements (10 samples per group; 10 measurements per sample). P value is < 0.001
